# Caffeic Acid, Reduced Glutathione, and Ferric Iron Addition Effects on the Redox Potential of Model Wine Solutions

**DOI:** 10.3390/molecules31071226

**Published:** 2026-04-07

**Authors:** William Jordan Wright, Dallas J. Parnigoni, Sean Kuster, James Nelson, Robert E. Coleman, L. Federico Casassa

**Affiliations:** 1 Wine and Viticulture Department, California Polytechnic State University, San Luis Obispo, CA 93407, USA; wjwright@calpoly.edu (W.J.W.); dallas@altacolina.com (D.J.P.); skuster@calpoly.edu (S.K.); 2 Department of Electrical and Computer Engineering, University of California, Davis, CA 95616, USA; jjnel@ucdavis.edu; 3 MeshVines, Sacramento, CA 95814, USA; 4 Department of Viticulture and Enology, Washington State University, Richland, WA 99354, USA; robert.coleman@wsu.edu

**Keywords:** caffeic acid, dissolved oxygen, glutathione, iron, oxidation–reduction potential, photo-Fenton

## Abstract

To further understand redox mechanisms occurring in wine, caffeic acid (CAF, 150 mg/L) and/or glutathione (GSH, 150 mg/L) were added to a model wine solution, followed by ferric iron (2 mg/L Fe(III), added as 10 mg/L Fe(III) chloride hexahydrate), while monitoring the oxidation–reduction potential (ORP, redox potential). Caffeic acid produced only modest ORP changes. In contrast, glutathione and caffeic acid + glutathione additions dropped the ORP from 243 mV and 238 mV, respectively, to the same post-addition value of 189 mV, suggesting that glutathione dictated the ORP, while caffeic acid showed no effect. The quinone of caffeic acid (assumed as changes in AU at 420 nm), was not detected, suggesting caffeic acid did not participate in oxidation reactions under wine conditions under superfluous amounts of dissolved oxygen (DO). After the addition of Fe(III), ORP increased to similar values across all treatments: 266 mV (FE), 269 mV (CAF), 284 mV (GSH), and 242 mV (CAF + GSH), suggesting that the Fe(II)/Fe(III) redox couple dominated the ORP electrode response. CAF + GSH produced the steepest ORP decline after the addition of Fe(III) chloride hexahydrate (β (slope of the ORP) = −0.7082), significantly steeper than FE (β = −0.3051; *p* = 0.0032) and GSH (β = −0.4643; *p* = 0.0496), suggesting synergistic radical quenching and metal redox cycling. Photo-Fenton-like reactions likely contributed to slight decreases in the ORP over time. In conclusion, glutathione strongly lowered the ORP, Fe(III) increased the ORP across treatments, and caffeic acid had minimal impact on the ORP under model wine conditions.

## 1. Introduction

Chemical oxidation influences the sensory properties and stability of wines. Uncontrolled oxidative processes in wine lead to the depletion of phenolic compounds [1], the loss of fruity aromas alongside the development of burnt, cooked, or aldehydic off odors [2,3,4], and ultimately a reduction in shelf life [5]. Because molecular oxygen (O_2_) affects both the chemical composition and sensory profiles of wine, understanding the mechanisms underlying chemical oxidation within the context of the complex chemical matrix of wine remains of interest for enology.

Model wine systems allow for the examination of the behavior of individual compounds and reactions under controlled conditions. Model wines typically consist of a solution of water, ethanol, and tartaric acid, with the pH adjusted to reflect that of actual wines [6,7]. These systems provide a simplified environment in which specific compounds can be selectively added, allowing oxidation and reduction reactions to be monitored without the confounding influence of other wine constituents.

Molecular oxygen is relatively stable on its own and does not react with most organic substances [8]. However, transition metals such as ferrous iron (Fe(II)) can activate O_2_ through a series of electron-transfer steps described by the Fenton reaction [9]. These reactions generate reactive oxygen species (ROS), including the hydroxyl radical (HO•). Furthermore, photo-Fenton chemistry accelerates the Fenton reaction by using light (*hv*; ≤410 nm) to photo-reduce ferric iron (Fe(III))-tartrate complexes back to Fe(II), thereby accelerating the rate-limiting step of the Fenton reaction without requiring peroxide and concurrently generating HO• [10]. The HO• radical can non-selectively oxidize virtually all wine components [11,12] and is therefore of significance when considering wine quality, stability, and shelf life.

The biosynthesis of phenolic compounds in plants aims to protect tissues from oxidative stress by scavenging free radicals formed during photosynthesis [13]. Among these, 3,4-dihydroxycinnamic acid, or caffeic acid, is a simple hydroxycinnamic acid derivative found in red wine at concentrations of 2 to 18 mg/L [14,15]. Caffeic acid was reported to be among the most readily available reducing agents in wine [8] due to its relative abundance and high propensity to donate electrons, making it a likely nucleophilic target to react with ROS. Caffeic acid has also been shown to form chelates with divalent metals such as Fe(II) and cupric cations [16], thereby limiting the participation of these metals in subsequent chemical reactions. The oxidation of phenolic compounds results in the formation of quinones, which can contribute to browning and promote further oxidation of compounds with lower reduction potentials, including odor-active volatiles [17,18].

**Figure 1:**
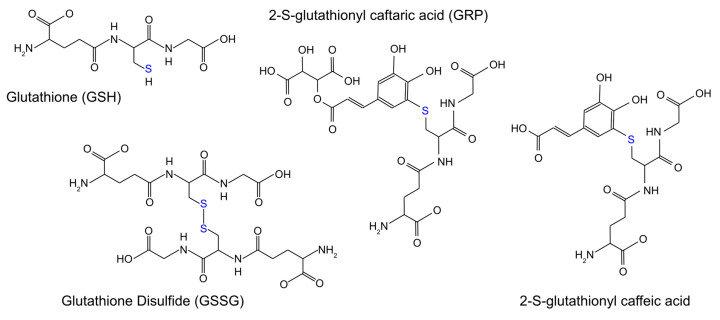
Chemical structures of glutathione, glutathione disulfide, 2-S-glutathionyl caftaric acid, and 2-S-glutathionyl caffeic acid. Blue fonts signify the sulfur center located on the original cysteine residue of glutathione.

**Figure 2:**
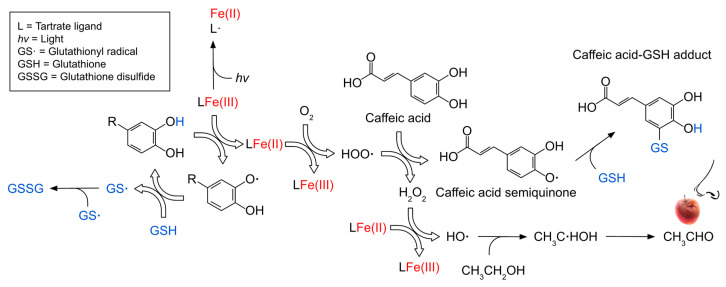
Proposed mechanism in model wine containing excess dissolved oxygen, ethanol, tartaric acid, Fe(III), caffeic acid, and glutathione, and formation of acetaldehyde (CH_3_CHO) from ethanol (CH_3_CH_2_OH). Blue and red fonts depict major effectors of the oxidation–reduction potential.

Glutathione (Figure 1), a tripeptide composed of L-glutamate, L-cysteine, and glycine, is commonly found in wine at concentrations ranging from 14 to 114 mg/L [19]. Glutathione is an effective reducing agent due to the highly oxidizable thiol group of its cysteine residue, which readily reacts with quinones [20] and scavenges ROS [21] (Figure 2). These reactions may proceed via two primary pathways: redox reactions, in which glutathione reduces quinones or ROS and is oxidized to glutathione disulfide (GSSG) (Figure 1) [22], thereby regenerating the corresponding phenol or quenching ROS; or else the spontaneous attack of the glutathione thiolate on quinones, resulting in the formation of stable phenol–glutathione adducts (Figure 2). These include, principally, the grape reaction product (GRP, 2-S-glutathionyl caftaric acid) (Figure 1) [23]. GRP is not a substrate for tyrosinase, a major polyphenol oxidase in grapes, highlighting this adduct’s role in stabilizing the oxidation cascade in wine, and thus preventing further browning [17]. It has additionally been proposed that 2-S-glutathionyl caffeic acid (Figure 1), formed from the reaction between glutathione thiolate and caffeic acid quinone, behaves similarly to GRP [23].

The oxidation–reduction potential (ORP) describes the tendency of redox-active species to accept or donate electrons (usually single electron transfer reactions) and therefore reflects a solution’s oxidative or reductive state [24]. Redox potential is measured using electrochemical sensors consisting of a working electrode, commonly platinum, that facilitates redox reactions, and a reference electrode that provides a stable voltage against which the working electrode is measured [25]. The standard hydrogen electrode (SHE) is a widely used reference electrode in electrochemistry; however, the silver/silver chloride (Ag/AgCl) reference electrode is less fragile and more commonly employed in industrial applications [26]. The potential of the SHE is approximately 220 mV higher than that of the Ag/AgCl electrode; accordingly, values measured against Ag/AgCl can be converted to SHE by adding 220 mV, and values measured against SHE can be converted to Ag/AgCl by subtracting 220 mV. Currently, there is no formal consensus in wine research regarding which reference electrode ORP values should be reported against.

Furthermore, disagreement exists regarding which species are most active at the surface of the platinum electrode and whether ORP represents a weighted sum of all ongoing redox processes or is dominated by a small number of key couples. It has been proposed that hydrogen peroxide (H_2_O_2_) generated from O_2_ via Fenton chemistry, along with the Fe(II)-tartrate/Fe(III)-tartrate, Cu(I)/Cu(II), and glutathione/GSSG couples, define the ORP [27]. In contrast, a recent study that assessed ORP using cyclic voltammetry suggested that ORP is primarily governed by the O_2_/ethanol redox couple [28]. Danilewicz et al. [28] highlight that SO_2_ and thiols, like glutathione, can bind to and poison metal electrodes, causing drift.

The present study aims to contribute to the understanding of the effects and consequences of the ORP in model wine solutions by examining the separate and combined effects of caffeic acid (a phenolic compound), glutathione (a thiol), and iron (a transition metal) in a model wine system lacking SO_2_ and containing superfluous dissolved oxygen.

## 2. Results and Discussion

### 2.1. Temperature

The overall mean temperatures in FE, CAF, GSH, and CAF + GSH were 29.4 °C, 29.5 °C, 29.5 °C, and 29.5 °C, respectively, during the time course of the experiments, with no differences among treatments (Supplementary Figure S1). Therefore, temperature was not considered a factor in influencing ORP measurements.

### 2.2. Dissolved Oxygen

**Figure 3:**
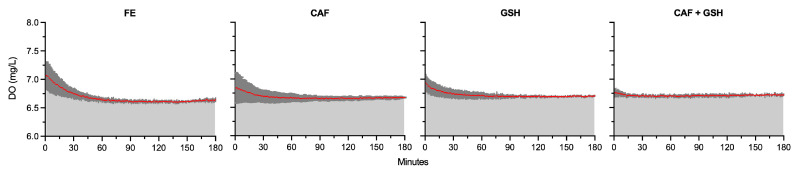
Dissolved oxygen (mg/L) of model wine solutions from 0 to 180 min. Each panel represents 3 replicates (n = 3). Red lines represent average dissolved oxygen, and dark grey shading represents the standard deviation.

Dissolved oxygen (DO) levels did not differ among treatments during the 0 to 60 min interval (Figure 3). Additionally, DO was always at superfluous levels during the time course of the experiment (Figure 3). During the 60 to 120 min interval, DO only marginally increased by 1.18% and 1.34% in GSH (6.70 mg/L) and CAF + GSH (6.71 mg/L), respectively, compared with FE (6.62 mg/L) (*p* < 0.0001). During the 120 to 180 min interval (*p* < 0.0001), DO was 0.74% lower in CAF (6.67 mg/L) relative to CAF + GSH (6.72 mg/L), and 0.72%, 1.15%, and 1.57% lower in CAF, GSH (6.69 mg/L), and CAF + GSH, respectively, compared with FE (6.62 mg/L). Overall, DO differences were exceedingly small and unlikely to influence ORP differences among treatments.

Coleman et al. described the ratio of O_2_ consumption to the oxidation of Fe(II) to Fe(III) under model wine conditions (pH 2.5 to 4.5; TA 4 g/L; Fe(II) 5 mg/L; DO 8.5 mg/L) as a 1:1 molar relationship [27]. The DO values in the present study (grand average of 6.70 mg/L) are close to the maximum O_2_ saturation typically observed in wine (8 mg/L) [1], indicating that O_2_ was present in excess and therefore readily available to oxidize transition metals.

### 2.3. Total Iron

**Figure 4:**
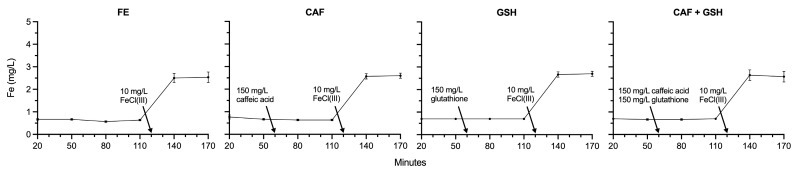
Measured total iron (mg/L) of model wine solutions from 20 to 170 min.

There were no differences in measured total iron among or within FE, CAF, GSH, and CAF + GSH from 20 to 110 min (Figure 4). Total iron increased from 0.63 mg/L to 2.50 mg/L in FE (*p* = 0.0009), 0.63 to 2.57 mg/L in CAF (*p* = 0.0001), 0.70 to 2.67 mg/L in GSH (*p* < 0.0001), and 0.70 to 2.63 mg/L in CAF + GSH (*p* = 0.0012) between 110 and 140 min, reflecting the addition of Fe(III) chloride hexahydrate, which is approximately 20.7% Fe(III) by weight, at 120 min. No differences in total iron were observed among treatments at the 110, 140, or 170 min time points.

These results show that iron, once dissolved into the model wine, remained in solution, and that no iron was lost following the addition of Fe(III) chloride hexahydrate. The reported values represent total iron measured in the model wine solutions and do not reflect the ratio of Fe(II) to Fe(III), a parameter known to have a substantial influence on the ORP [29]. Given the dual role of iron as both a reducing and oxidizing agent through redox cycling between Fe(II) and Fe(III), its persistence in the model wine suggests not only an immediate but also a sustained ability to influence the ORP and, consequently, wine stability. Small amounts of residual iron were detected at the 20, 50, 80, and 110 min time points, likely originating from impurities in the laboratory-grade potassium bitartrate, tartaric acid, or ultrapure water used in preparing the model wine solution. Nonetheless, a methodological limitation of the present study is that Fe speciation (Fe(II)/Fe(III)) was not determined. Follow up studies should strive to measure Fe speciation by techniques such as capillary electrophoresis (CE) and inductively coupled plasma (ICP) spectroscopic detection or ICP-MS.

### 2.4. Absorbance Units at 280 nm, 325 nm, and 420 nm

**Figure 5:**
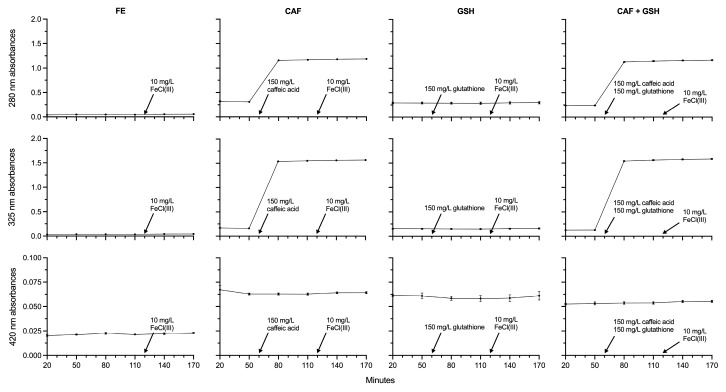
Absorbances (280 nm, 325 nm, and 420 nm) of model wine solutions from 20 to 170 min.

Spectrophotometric scans at 280 nm, 325 nm, and 420 nm were used to measure total phenolics [30], caffeic acid [31], and browning [32,33] in the model wine solution, respectively. Absorbance at 280 nm did not change between 20 and 170 min in FE (*p* = 0.1000) or GSH (*p* = 0.9990) (Figure 5). In contrast, 280 nm absorbance increased by 275% in CAF (*p* < 0.0001) and by 369% in CAF + GSH (*p* < 0.0001) between 50 and 80 min.

In CAF, absorbance at 280 nm (*p* < 0.0001) increased slightly, yet significantly, between 80 and 110 min (0.81%), 110 and 140 min (0.93%), and 140 and 170 min (0.60%). In CAF + GSH, no differences in 280 nm absorbance were observed among the 80, 110, 140, and 170 min time points (*p* = 0.1302). At 80 min, 280 nm absorbance in CAF and CAF + GSH was 2303% and 2243% higher than in FE (*p* < 0.0001), 2578% and 2527% higher at 110 min (*p* < 0.0001), 2090% and 2055% higher at 140 min (*p* < 0.0001), and 2036% and 1999% higher at 170 min (*p* < 0.0001). Similarly, 280 nm absorbance in CAF and CAF + GSH exceeded that of GSH by 303% and 293% at 80 min, 308% and 301% at 110 min, 301% and 449% at 140 min, and 298% and 292% at 170 min.

There were no statistical differences in 325 nm absorbance in GSH from 20 to 170 min (*p* = 0.9861), and the only change observed in FE was a 30% difference between the 20- and 170-min time points (*p* = 0.0157) (Figure 5). In contrast, large increases in 325 nm absorbance occurred between 50 and 80 min in CAF (867%, *p* < 0.0001) and CAF + GSH (1108%, *p* < 0.0001).

In CAF, absorbance at 325 nm increased by 1.40% between 80 and 140 min and by 1.10% between 110 and 170 min (*p* = 0.0015). No differences in 325 nm absorbance were observed in CAF + GSH among the 80, 110, 140, and 170 min time points (*p* = 0.1111). At 80 min, 325 nm absorbance in CAF and CAF + GSH was 4177% and 4182% higher than in FE (*p* < 0.0001), 4490% and 4522% higher at 110 min (*p* < 0.0001), 3719% and 3757% higher at 140 min (*p* < 0.0001), and 3620% and 3659% higher at 170 min (*p* < 0.0001). Similarly, 325 nm absorbance in CAF and CAF + GSH exceeded that of GSH by 930% and 932% at 80 min, 945% and 953% at 110 min, 909% and 919% at 140 min, and 890% and 900% at 170 min.

No statistical differences in absorbance at 420 nm were observed over time (20 to 170 min) within FE (*p* = 0.2063), CAF (*p* = 0.6252), GSH (*p* = 0.9383), or CAF + GSH (*p* = 0.5757) (Figure 5). From 20 to 140 min, 420 nm absorbance was highest in CAF, followed by CAF + GSH, both of which were higher than FE; GSH was statistically similar to CAF and CAF + GSH, but higher than FE. By 170 min, CAF, GSH, and CAF + GSH were statistically similar to each other, though all remained higher than FE.

Absorbance at 280 nm, used to estimate total phenolic content in wine [30], showed no changes over time following the addition of caffeic acid (i.e., at the 80, 110, 140, and 170 min time points). Absorbance at 325 nm, which more specifically reflects caffeic acid content [31], also showed no differences between CAF and CAF + GSH over time. These results suggest that caffeic acid neither oxidized in the CAF solution, nor formed adducts with glutathione in the CAF + GSH solution.

Analysis of absorbance at 420 nm, a metric for assessing browning [32,33], also suggested no signs of formation of brown pigments, such as quinones, in any treatment during the experiment.

Overall, absorbances at 280, 325, and 420 nm reflected the addition of caffeic acid at 60 min but did not indicate the formation of brown pigments such as caffeic acid quinone or other phenol-derived oxidation products. The absorbance of FE at all wavelengths was consistently lower than that of CAF, GSH, and CAF + GSH. These differences most likely reflect variation in the tartaric acid content of the model wine solutions due to post-preparation precipitation. However, the trends remained consistent.

### 2.5. Oxidation–Reduction Potential

As during the 0 to 60 min interval, the ORP in CAF (255 mV) was 8% and 12% higher than in CAF + GSH (237 mV) and FE (228 mV), respectively *(p =* 0.0032). Any differences in the ORP among treatments during the 0 to 60 min interval, prior to additions, were likely due to tartrate precipitation in the model wine solutions following their preparation. However, these initial differences in the ORP were relatively minor and reflect the sensitivity of ORP measurement towards the formation of Fe(III)–tartrate complexes. Between 60 and 120 min (*p* < 0.0001), the ORPs of GSH (189 mV) and CAF + GSH (189 mV) were 25% lower than that of CAF (252 mV). Over the same interval, the ORP of FE (222 mV) was 18% higher than GSH and CAF + GSH, but 12% lower than CAF. During the 120 to 180 min interval (*p* = 0.0001), the ORP of GSH (284 mV) was 6%, 7%, and 17% higher than that of CAF (269 mV), FE (266 mV), and CAF + GSH (242 mV), respectively. CAF and FE were also 11% and 10% higher than CAF + GSH.

The mean ORP of GSH (*p* = 0.0006) and CAF + GSH (*p* = 0.0013) decreased significantly between the 0 to 60 and 60-to-120 min intervals, dropping from 243 mV to 189 mV (a 54 mV or 22% decrease) and from 237 mV to 189 mV (a 48 mV or 20% decrease), respectively. This decrease was not observed in FE (*p* = 0.0563) or CAF (*p* = 0.1437). There were also significant increases in the mean ORP between the 60 to 120 and 120 to 180 min intervals for FE (*p* = 0.0003), CAF (*p* = 0.0001), GSH (*p* < 0.0001), and CAF + GSH (*p* = 0.0009). The ORP increased from 222 mV to 266 mV in FE (44 mV; 20%), from 252 mV to 269 mV in CAF (17 mV; 7%), from 189 mV to 284 mV in GSH (95 mV; 50%), and from 189 mV to 242 mV in CAF + GSH (53 mV; 28%).

The ORP slope (β) of CAF + GSH (β = −0.7082) during the 120 to 180 min interval was 132%, 46%, and 53% steeper than the slopes of FE (β = −0.3051), CAF (β = −0.4856), and GSH (β = −0.4643), respectively (*p* = 0.0051). However, significant differences were found only between CAF + GSH and FE (*p* = 0.0032) and between CAF + GSH and GSH (*p* = 0.0496).

**Figure 6:**
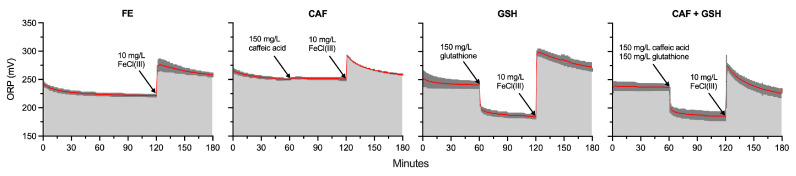
Oxidation–reduction potential (mV) of model wine solutions from 0 to 180 min. Each panel represents 3 replicates (n = 3). Red lines represent average ORP, and dark grey shading represents standard deviation. Average ORP (mV) was calculated as the average of ORP values.

Four main takeaways can be derived from the ORP data (Figure 6). First, the resting ORP, defined as “a stable redox state reached following a short-term disruption in the ORP” [34], at roughly 250 mV (Ag/AgCl), was relatively unaffected by an excess addition of caffeic acid in the presence of trace amounts of transition metals and superfluous amounts of DO. Previous studies using cyclic voltammetry and spectroelectrochemistry have shown that caffeic acid oxidizes rapidly at pH 4.0 to a short-lived (10^−4^ s) semiquinone and then to the corresponding quinone [35]. Hapiot et al. also reported that this reaction is reversible, suggesting that the quinone can be reduced back to its phenolic form under sufficiently reductive (low ORP) conditions [35]. Under the conditions of the present study, it is possible to speculate that the phenol/quinone half-cell potential of caffeic acid under model wine conditions lies near or slightly above 255 mV (Ag/AgCl), which was the resting ORP of the model wine system at the time caffeic acid was added. Consequently, the expected impact of caffeic acid on the ORP during actual winemaking conditions would be expected to be modest, consistent with the observations in this study. Previous works have suggested that phenolics do not contribute to the ORP observed in wine [36,37,38,39]. Despite the findings of the present study and supporting works, the influence of phenolic compounds, especially those containing catechol or pyrogallol groups, on the ORP remains understudied in both fermenting and finished wines.

Secondly, a substantial addition of glutathione or a mixture of caffeic acid and glutathione rapidly lowered the ORP by approximately 50 mV. The antioxidant properties of glutathione in wine are well-documented [40,41,42], as is the dominance of the glutathione/GSSG couple at the electrode surface in model wine solutions [38]. However, the immediate ORP response to glutathione addition in such a system has not previously been reported. As expected, the addition of glutathione decreased the ORP in both GSH and CAF + GSH. Notably, the resting ORPs of GSH and CAF + GSH treatments (initially 244 mV and 238 mV, respectively) converged to the same value after glutathione addition (189 mV). This indicates that (i) caffeic acid did not influence the ORP when in the presence of glutathione, and (ii) the ORP dropped to a consistent, glutathione-determined resting value regardless of the initial ORP. In agreement with Nelson et al., the glutathione/GSSG half-cell reaction likely dominates the ORP in the absence of significant transition metal–tartrate complexes [38].

Thirdly, in all treatments, the addition of Fe(III) increased the ORP to similar values despite the treatments starting from different resting ORPs. This suggests that the Fe(II)/Fe(III) couple dominated the electrode response in the presence of caffeic acid, glutathione, both, or neither. Following Fe(III) addition, the ORP slope (β) was steepest in CAF + GSH, indicating the fastest return to a new resting ORP. This observation was confirmed by a one-way Analysis of Variance of the area under the curve (AUC) of the ORP for the 120 to 180 min interval, where only CAF + GSH differed significantly from FE (*p* = 0.0001). It is hypothesized that the combination of caffeic acid and glutathione likely lowered ORP through a synergistic mechanism involving radical quenching, Fe(II) regeneration, and inhibition of Fe(II) oxidation. However, the present study quantified total iron. Future studies should quantify Fe(II) and Fe(III) separately, rather than relying solely on total iron measurements, as this would provide deeper insight into how the Fe(II)/Fe(III) ratio affects the ORP.

Fourthly, photo-Fenton reactions likely occurred in all treatments and must be considered when interpreting ORP behavior (Figure 2). Further experimental data (Supplementary Figure S2) indicated that photo-Fenton reactions, catalyzed by *hv*, may have lowered the ORP by rapidly reducing Fe(III)–tartrate complexes to Fe(II) while simultaneously oxidizing the tartrate ligand [10]. If indeed the photo-Fenton process occurred, it contributed to the observed reducing power (i.e., the decline in the β of the ORP), not the oxidizing power of the solution. Therefore, photo-Fenton-induced reduction of Fe(III) to Fe(II) contributed to the observed ORP response, while the oxidation of the tartrate ligand did not. Photo-Fenton should be minimized in future experiments by protecting wine or model wine solutions from *hv* exposure.

## 3. Materials and Methods

### 3.1. Model Wine Formulation

Ultrapure water (860 mL) was added to a 1500 mL glass beaker and placed on a Corning PC-420D stirring hot plate (Corning, NY, USA) set to stir at 200 rpm. The model wine solution temperature was kept below 50 °C throughout preparation. Laboratory-grade potassium bitartrate (5 g; Ward’s Science, Rochester, NY, USA) and reagent-grade tartaric acid (5 g; Fisher Scientific, Waltham, MA, USA) were added to the solution. After complete dissolution, the heat was turned off, and 140 mL of 190-proof spectrophotometric-grade ethanol (Thermo Scientific, Waltham, MA, USA) was incorporated. Once homogenized, the pH was measured with an Orion Star A211 m (Thermo Scientific, Waltham, MA, USA) and adjusted to pH 3.50 using sodium hydroxide pellets (VWR, Radnor, PA, USA). The resulting model wine solution had a pH of 3.50, titratable acidity (TA) of 5.13 g/L, and an alcohol by volume (ABV) of 13%. These values were measured using an Orion Star A211 m (Thermo Scientific, Waltham, MA, USA), a HI 901 W automatic titrator (Hanna Instruments, Woonsocket, RI, USA), and an Alcolyzer Wine M alcohol meter (Anton Paar, Graz, Austria), respectively.

### 3.2. Experimental Setup

A water bath was placed on an Isotemp Stirrer (Fisher Scientific, Waltham, MA, USA) and set to 30 °C using a Precision Cooker 3.0 (Anova Applied Electronics, San Francisco, CA, USA). One liter of model wine solution was poured from a sealed 1000 mL jar into a 1500 mL beaker and positioned within the water bath. The stirrer was adjusted to 200 rpm. An RC-5+ temperature sensor (Elitech Technology, San Jose, CA, USA), an EasyFerm Plus (Ag/AgCl) ORP sensor (Hamilton Company, Reno, NV, USA), and a NOMASense O_2_ P300 DO sensor (Vinventions, Thimister-Clermont, Belgium) were then placed into the model wine solution, and all devices were set to begin data collection.

**Figure 7:**
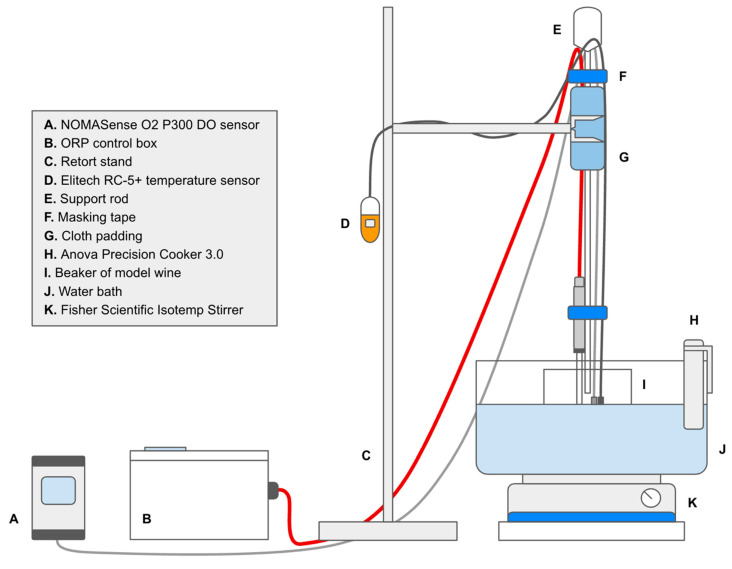
Experimental design and setup of dissolved oxygen and oxidation–reduction sensor probes in model wine solutions.

Prior to experimentation, the ORP sensor was calibrated using a +271 mV buffer solution and a Wi 1 G adapter connected to the ArcAir application (Hamilton Company, Reno, NV, USA). The sensor was connected by a VP8 cable (Hamilton Company, Reno, NV, USA) to an ORP control box (MeshVines, Davis, CA, USA) (Figure 7, Supplementary Figure S3).

After 60 min of data collection, 150 mg/L of caffeic acid (Tokyo Chemical Industry, Tokyo, Japan), 150 mg/L of glutathione (Belle Chemical, Billings, MT, USA), or 150 mg/L of each compound were added to the model wine solution. The CAF treatment received 150 mg/L caffeic acid, GSH received 150 mg/L glutathione, and CAF + GSH received both additions. The FE treatment received no caffeic acid or glutathione. After 120 min of data collection, 10 mg/L of reagent-grade Fe(III) chloride hexahydrate (Sigma Aldrich, St. Louis, MO, USA) was added to all treatments. Samples were collected at 20, 50, 80, 110, 140, and 170 min for enzymatic and spectrophotometric analyses. Data collection concluded at 180 min. Each treatment consisted of three replicates (n = 3).

### 3.3. Analysis

#### 3.3.1. Spectrophotometric Analysis

Wavelengths were measured at 280 nm, 325 nm, and 420 nm using a Cary 60 UV-Vis spectrophotometer with an 18-position auto-sampler (Agilent Technologies, Santa Clara, CA, USA). Iron was analyzed spectrophotometrically using a two-reagent differentiation method, with absorbance measured at 560 nm. Analyses were performed using a commercially available kit (Biosystems, Barcelona, Spain) and a SPICA analyzer (Admeo, Angwin, CA, USA) at 20, 50, 80, 110, 140, and 170 min after the start of data collection.

#### 3.3.2. Statistical Analysis and Data Visualization

Excel (Microsoft, Redmond, WA, USA) was used for data organization, and JMP (JMP 18.2.1. Statistical Discovery LLC, Cary, NC, USA) was used for all statistical analyses. To avoid pseudo replication, the mean of each replicate across time was used for primary analysis. Datasets with two treatments were analyzed using Welch’s two-sample *t*-test (two-sided, α = 0.05) on per-replicate means. Datasets with three treatments were analyzed using one-way Analysis of Variance to test for differences among per-replicate means, followed by the Tukey–Kramer honestly significant difference (HSD) post hoc test to determine which treatments differed. Prism (GraphPad Software Inc., La Jolla, CA, USA) was used to create all figures except Figure 1, Figure 2 and Figure 7, which were prepared using Google Slides (Google, Mountain View, CA, USA).

## 4. Conclusions

The present study investigated the separate and combined additions of caffeic acid, glutathione, and Fe(III) on the fate and evolution of the ORP in a highly oxygenated model wine system. In such solutions containing tartaric acid and transition metals, no significant changes in ORP were observed following the addition of caffeic acid, despite the known oxidizability of this compound. This finding is consistent with the reported ORP of the phenol/quinone redox couple, which is close to the resting ORP that was observed in the present model wine system. In contrast, glutathione quickly lowered the ORP by approximately 50 mV and emerged as a potentially powerful reductant and effector of the ORP in wines. Both GSH and CAF + GSH converged to the same resting ORP after glutathione addition, suggesting that glutathione dominated the ORP response and drove the system single-handedly towards a glutathione-determined value. Despite high dissolved oxygen, absorbance data showed no indication of permanent quinone formation in the form of browning over the two hours of the experiment, implying that caffeic acid did not oxidize in these model wine solutions.

After the addition of Fe(III) chloride hexahydrate, the ORP increased to similar values across all treatments, suggesting that Fe(III), which is the predominant oxidized iron species in wine matrices, overrode the influence of caffeic acid and glutathione and dominated the ORP. CAF + GSH showed the steepest decline in the ORP when returning toward a resting state, implying that the combination of caffeic acid and glutathione, likely through radical quenching and transition-metal cycling, exerted stronger reducing power than either compound alone.

Overall, the results indicate that glutathione strongly decreases the ORP (increasing reducing power), Fe(III) additions increase the ORP (increasing oxidative power and overriding glutathione effects), and caffeic acid has no measurable impact on the ORP in conditions where the resting ORP lies near the reduction potential of the phenol/quinone couple. These results can likely be extrapolated to actual wine conditions. For example, excess dissolved oxygen in a wine or wine-like system may not cause oxidation if transition metals do not have a chemical pathway to quickly recirculate back to their reduced form. However, actual wines contain a host of reductants able to perform such recirculation; if these reductants and other ligands (such as tartaric acid) can be selectively removed, then wine oxidation can be theoretically stopped. In addition, because we suggest that photo-Fenton reactions likely caused a gradual decrease in the ORP through *hv*-catalyzed reduction of Fe(III) to Fe(II), implications for wines packaged in transparent glass bottles can also be derived. Future studies may aim at replicating this experimental design with different ethanol levels, in the absence of oxygen and *hv*, with additions of Fe(II), and/or in the presence of other transition metals (alone or in combination), while quantifying transition metal speciation, and with additions of other thiols, other than glutathione, typically found in wine.

## Data Availability

The original contributions presented in this study are included in the article/Supplementary Materials. Further inquiries can be directed to the corresponding author.

## Supplementary Materials

The following supporting information can be downloaded at https://www.mdpi.com/article/10.3390/molecules31071226/s1, Figure S1: Temperature (°C) of model wine solutions from 0 to 180 min. Each panel represents 3 replicates (n = 3). Red lines represent average temperature, and dark grey shading represents the standard deviation; Figure S2: Oxidation–reduction potential (mV) of model wine solution from 0 to 180 min. Represents 1 replicate (n = 1); Figure S3: Photograph of setup during execution of the experiment.

## Abbreviations

The following abbreviations are used in this manuscript:

ABV	Alcohol by volume
Ag/AgCl	Silver/silver chloride
AUC	Area under the curve
DO	Dissolved oxygen
GRP	Grape reaction product
GS•	Glutathionyl radical
GSH	Glutathione
GSSG	Glutathione disulfide
H_2_O_2_	Hydrogen peroxide
HO•	Hydroxyl radical
HSD	Honestly significant difference
*hv*	Light
O_2_	Molecular oxygen
ORP	Oxidation–reduction potential
ROS	Reactive oxygen species
SHE	Standard hydrogen electrode
SO_2_	Sulfur dioxide
TA	Titratable acidity
