# Supplementary material for: Caffeic Acid, Reduced Glutathione, and Ferric Iron Addition Effects on the Redox Potential of Model Wine Solutions
Source: Molecules. 2026 Apr 7;31(7):1226. doi: 10.3390/molecules31071226 (PMC13074730; doi:10.3390/molecules31071226)
Supplement: Supplementary file 1 [file molecules-31-01226-s001.zip › molecules-4186942-supplementary.pdf]

Supplementary Materials

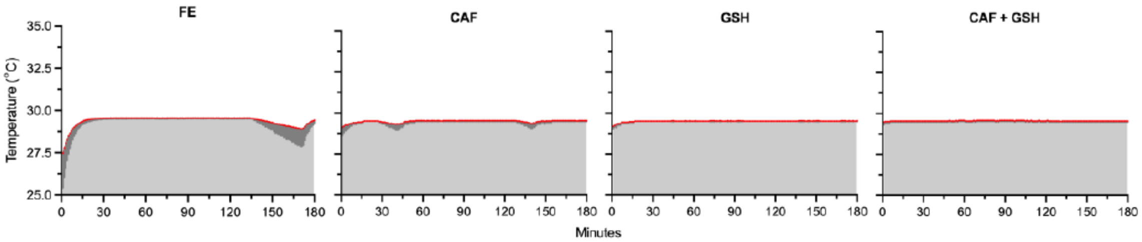

Supplementary Figure S1. Temperature (ºC) of model wine solutions from 0 to 180 min. Each panel represents 3 replicates (n = 3). Red lines represent average temperature, and dark grey shading represents the standard deviation.

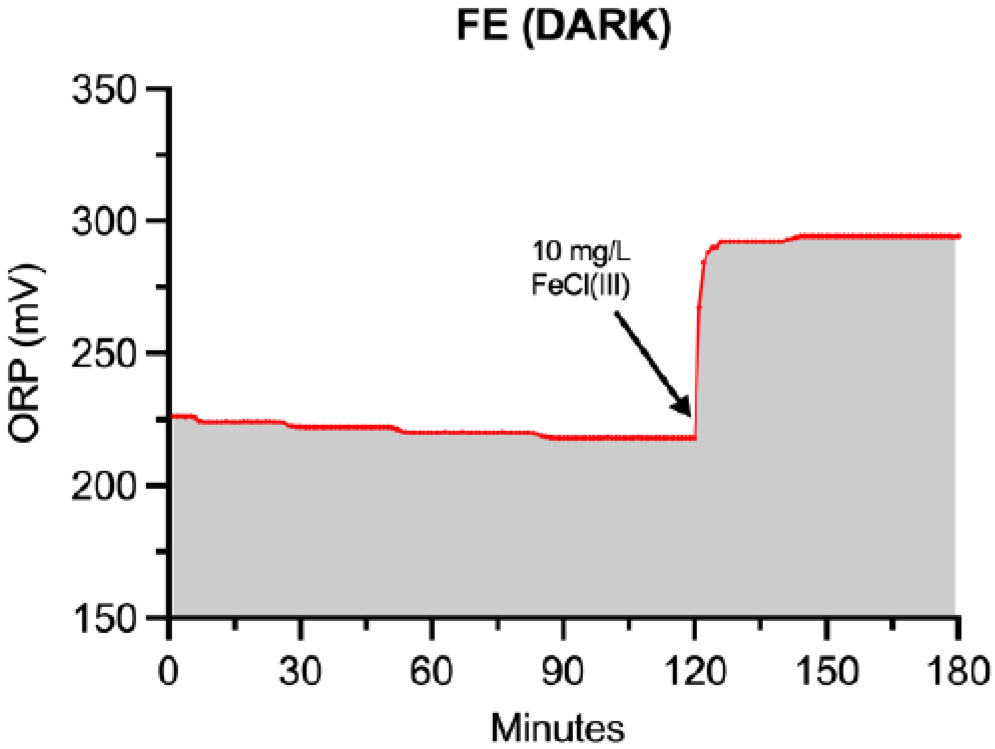

Supplementary Figure S2. Oxidation-reduction potential (mV) of model wine solution from 0 to 180 min. Represents 1 replicate (n = 1).

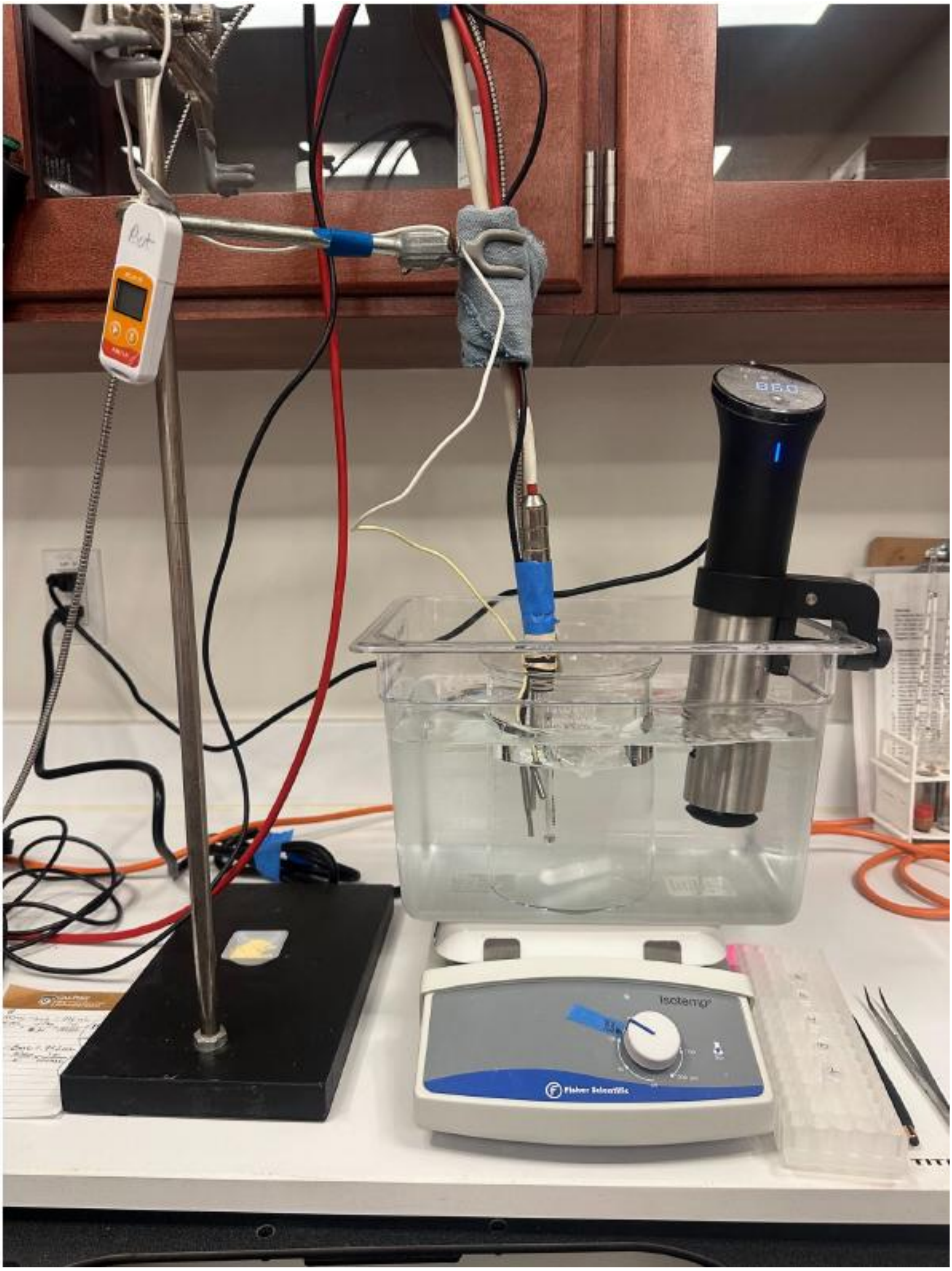

Supplementary Figure S3. Photograph of setup during execution of the experiment.
